# Craniopharyngioma – A Childhood and Adult Disease with Challenging Characteristics

**DOI:** 10.3389/fendo.2012.00080

**Published:** 2012-07-04

**Authors:** Hermann L. Müller

**Affiliations:** ^1^Department of Pediatrics, Klinikum Oldenburg gGmbHOldenburg, Germany

Craniopharyngiomas are rare embryogenic malformations of the sellar area with low-grade histological malignancy. Despite high survival rates (87–95% in recent series), quality of life is frequently impaired in long-term survivors due to sequelae caused by the anatomical proximity of the tumor to the optic nerve, pituitary gland, and hypothalamus – in many cases even entanglement with the hypothalamus (Cohen et al., 2011; Müller, 2011; Zoicas and Schöfl, 2012). One of the most serious quality of life complications of craniopharyngioma is hypothalamic obesity or so-called “hypothalamic syndrome,” a problem that can manifest before and/or after treatment. Novel insights into mechanisms of neuroendocrine satiety regulation and the pathogenic relevance of the autonomous nervous system are expected to facilitate future therapeutic approaches for hypothalamic syndrome (Elfers and Roth, 2011; Lustig, 2011; Roth, 2011).

For the time being, current treatment options for craniopharyngioma patients suffering from hypothalamic syndrome are limited, the most effective to date being bariatric surgery. Bariatric surgery is tolerable and effective in weight-reduction for severely obese adult craniopharyngioma patients (Bingham et al., 2012), but considered intrusive and therefore controversial for younger patients. A substantial improvement in prognosis of craniopharyngioma will require the development of risk adapted neurosurgical (Flitsch et al., 2011; Bartels et al., 2012; Puget, 2012; Trippel and Nikkhah, 2012) and radiooncological (Kortmann, 2011) treatment strategies in a multidisciplinary approach. Recent multicenter cooperation in this area has already led to beneficial results.

The consequences of both the surgical treatment and post-surgical management of the disease are as complicated and hypothalamic-intertwined as the tumors themselves. Risk adapted surgical strategies at initial diagnosis should aim at a maximal degree of resection, respecting the integrity of optical and hypothalamic structures in order to prevent severe sequelae and therein minimize consequences that could negatively exacerbate patient quality of life. Because initial hypothalamic tumor involvement typically has an *a priori*, life-long effect on the clinical course (Müller, 2011) experienced by the patient, childhood, and adult onset craniopharyngioma should be recognized as chronic diseases requiring constant monitoring of the consequences and developing medical resources for treatment in order to provide not only optimal quality of life for patients, but also to garner additional information with the intent of minimizing what at present are severe consequences of both the disease and its treatment.

## References

[B1] Bartels U. Laperriere N. Bouffet E. Drake J. (2012). Intracystic therapies for cystic craniopharyngioma in childhood. Front. Endocrinol. (Lausanne) 3:39 10.3389/fendo.2012.00039 22654864 PMC3356106

[B2] Bingham N. C. Rose S. R. Inge T. H. (2012). Bariatric surgery in hypothalamic obesity. Front. Endocrinol. (Lausanne) 3:23 10.3389/fendo.2012.00023 22649412 PMC3355900

[B3] Cohen M. Guger S. Hamilton J. (2011). Long term sequelae of pediatric craniopharyngioma – literature review and 20 years of experience. Front. Endocrinol. (Lausanne) 2:81 10.3389/fendo.2011.00081 22645511 PMC3355823

[B4] Elfers C. T. Roth C. L. (2011). Effects of methylphenidate on weight gain and food intake in hypothalamic obesity. Front. Endocrinol. (Lausanne) 2:78 10.3389/fendo.2011.00078 22649386 PMC3355874

[B5] Flitsch J. Müller H. L. Burkhardt T. (2011). Surgical strategies in childhood craniopharyngioma. Front. Endocrinol. (Lausanne) 2:96 10.3389/fendo.2011.00096 22645514 PMC3355821

[B6] Kortmann R.-D. (2011). Different approaches in radiation therapy of craniopharyngioma. Front. Endocrinol. (Lausanne) 2:100 10.3389/fendo.2011.00100 22654836 PMC3356005

[B7] Lustig R. H. (2011). Hypothalamic obesity after craniopharyngioma: mechanisms, diagnosis, and treatment. Front. Endocrinol. (Lausanne) 2:60 10.3389/fendo.2011.00060 22654817 PMC3356006

[B8] Müller H. L. (2011). Diagnostics, treatment, and follow-up in craniopharyngioma. Front. Endocrinol. (Lausanne) 2:70 10.3389/fendo.2011.00096 22654824 PMC3356030

[B9] Puget S. (2012). Treatment strategies in childhood craniopharyngioma. Front. Endocrinol. (Lausanne) 3:64 10.3389/fendo.2012.00064 22679439 PMC3367313

[B10] Roth C. L. (2011). Hypothalamic obesity in patients with craniopharyngioma: profound changes of several weight regulatory circuits. Front. Endocrinol. (Lausanne) 2:49 10.3389/fendo.2011.00049 22654811 PMC3356147

[B11] Trippel M. Nikkhah G. (2012). Stereotactic neurosurgical treatment options for craniopharyngioma. Front. Endocrinol. (Lausanne) 3:63 10.3389/fendo.2012.00063 22654877 PMC3356096

[B12] Zoicas F. Schöfl C. (2012). Craniopharyngioma in adults. Front. Endocrinol. (Lausanne) 3:46 10.3389/fendo.2012.00046 22654868 PMC3356097

